# Integrating mental health into climate change education to inspire climate action while safeguarding mental health

**DOI:** 10.3389/fpsyg.2023.1298623

**Published:** 2024-01-08

**Authors:** Jessica Newberry Le Vay, Alex Cunningham, Laura Soul, Heena Dave, Leigh Hoath, Emma L. Lawrance

**Affiliations:** ^1^Institute of Global Health Innovation, Imperial College London, London, United Kingdom; ^2^Grantham Institute—Climate Change and the Environment, Imperial College London, London, United Kingdom; ^3^Anna Freud, London, United Kingdom; ^4^Natural History Museum, London, United Kingdom; ^5^Climate Adapted Pathways for Education, UK Wide, United Kingdom; ^6^Teacher Development Trust, London, United Kingdom; ^7^School of Education, University of Stirling, Stirling, Scotland, United Kingdom; ^8^Faculty of Social Sciences and Education, Leeds Trinity University, Leeds, United Kingdom; ^9^Mental Health Innovations, London, United Kingdom

**Keywords:** children and young people (CYP), climate change education (CCE), climate change, whole school approach, climate distress, climate action, mental health and wellbeing

## Abstract

Climate change is the greatest threat humanity faces, and puts at risk the mental health and wellbeing of children and young people. Climate change education must equip children and young people with the knowledge, skills and resilience to live in an uncertain future, sustainably take relevant climate action and work in climate careers. As attention on climate change education grows, this is a critical moment for the mental health community to ensure mental health and wellbeing considerations are embedded. Critically, appropriate integration of mental health can enable these very necessary goals of equipping children and young people to live and work in a future where climate change looms large. This paper explores why promoting good mental health and wellbeing and building psychological resilience can help achieve climate change education outcomes, and why not doing so risks harming children and young people’s mental health. It also explores how integrating discussions about emotions, mental health, and coping strategies within climate change education can be a route into wider discussions about mental health, to support children and young people in the context of rising mental health needs. Learning from an existing approach to promoting good mental health and wellbeing in schools (the ‘whole school approach’) provides the opportunity to explore one avenue through which such an integrated approach could be implemented in practice. Identifying appropriate mechanisms to integrate mental health into climate change education will require co-design and research with educators and young people, and addressing systemic barriers facing the schools sector.

## 1 Introduction

Climate change education (CCE) is critical for preparing children and young people (CYP) for their future and as future leaders. There is a clear and growing need for education that equips them with the knowledge, tools, resilience and agency to live in an uncertain future, take climate action and work in climate careers. Climate change is the biggest threat humanity faces (Calvin et al., 2023; World Health Organization, 2023), and it is imperative that education tells the truth about the situation. Indeed not doing so is a cause of distress for many young people when they realize their education has left them ill-prepared for the future they face (Diffey et al., 2022; British Science Association, 2023). Doing so without appropriate support, however, also risks distress; having an awareness of and learning about climate change can raise distress that, without coping strategies, can have harmful impacts on mental health and wellbeing (Godsmark, 2020; Ojala et al., 2021; British Science Association, 2023; Ojala, 2023). Creating space within CCE for CYP to understand and reflect on their psychological responses to climate change and the links with narratives they hold and are told, will help enable them to act, and act sustainably. Strengthening their ability to process and express challenging emotion, and develop agency and resilience, can also help mental health more broadly, and can pave the way to normalizing wider mental health discussions. Integrated approaches to climate change and mental health education may have win-wins for both. While literature has started to explore these links (Cantell et al., 2019; Bryan, 2020; Godsmark, 2020; Pihkala, 2020a; Ojala, 2023), this is yet to be fully understood or implemented.

Children and young people in the Keyes et al. (2023) are also facing rising mental health needs, exacerbated by multiple intersecting crises—including climate change (NHS Digital, 2023). Climate change threatens mental health in a range of ways, which CYP are particularly vulnerable to (Sanson et al., 2019; Hickman et al., 2021; Léger-Goodes et al., 2022). Directly experiencing climate impacts and the on-going disruption to communities can lead to new cases or worsened symptoms of mental health challenges such as depression, anxiety, post-traumatic stress disorder, and substance abuse (Berry et al., 2010; Hayes et al., 2018; Lawrance et al., 2022). Awareness of and learning about climate change and witnessing inaction of decision makers is also linked to strong emotional responses and distress. Such responses are simultaneously caring, healthy, valid *and* stressors that can create or exacerbate mental health challenges (Verplanken and Roy, 2013; Clayton, 2020; Ogunbode et al., 2021; Ojala et al., 2021). Some psychological responses to climate change, such as overwhelm and denial, can be barriers to engaging with climate action (Mayer and Smith, 2019). The impacts of climate change on mental health can limit the capacity of individuals, communities and systems to be able to take climate action. A vicious cycle between the climate crisis and mental health crisis therefore exists. There is potential, however, for a vicious cycle to become a virtuous one. Some psychological responses can be motivating for climate action, such as hope and anger (Vercammen et al., 2023). Taking climate action can itself have benefits for mental health; pro-environmental behaviors and collective climate action can help CYP build agency, hope and community connection, and be protective against climate-related distress (Schwartz et al., 2022). It is important to note, however, that engaging in climate action when not appropriately supported can also harm mental health, for example by leading to burnout (Godden et al., 2021; Diffey et al., 2022; Conner et al., 2023). At a societal level, climate action (such as safe, secure, and energy efficient housing) can help foster conditions needed for good mental health (Zawadzki et al., 2020; Lawrance et al., 2022; Schwartz et al., 2022). Climate action and mental health action can align to strengthen both mental health and a safer climate future.

Young people want solutions-focused CCE, opportunities to take climate action (British Science Association, 2023; InterClimate Network, 2023), and for mental health to be integrated into learning about the climate crisis (Diffey et al., 2022). Most are not yet experiencing this. In the United Kingdom, 6 in 10 young people report not feeling part of decisions about what climate action their school should take, or discussing climate change solutions in class (InterClimate Network, 2023). Current approaches to CCE can contribute to young people’s sense of climate anxiety and not inspire hope (British Science Association, 2023). Attention on CCE is growing (Department for Education, 2022), as are calls from educators and students for improved approaches, for example that embed climate change across the whole curriculum (Teach The Future, 2021). Developing new approaches to CCE that can respond to these needs offers an opportunity to integrate better promotion of good mental health and wellbeing in the context of CCE in schools. Learning from and exploring adaptation of existing frameworks may be a useful first step. For example, the whole school approach (WSA) to supporting CYP mental health and wellbeing (HM Government, 2021). This means that mental health and wellbeing is embedded into the heart of a school’s ethos, curriculum, and culture. It includes collaborating with students, parents and carers, mental health experts, and being championed by school leadership. Success is evident when the mental health and wellbeing of students, staff and the wider school community is prioritized, and they know when and how to access further support when needed.

This paper explores ways in which the goals of CCE and safeguarding CYP mental health are aligned, what this could look like in practice using th example of a WSA, and is a call to action for the mental health community, climate change community, and education sector to come together to work toward these goals. While illustrated using the context in England, this paper draws on learnings from other countries and aims to have relevance to other contexts.

## 2 Safeguarding and promoting mental health and wellbeing through climate change education

Children and young people are particularly vulnerable to experiencing harmful mental health and wellbeing impacts of climate change (Sanson et al., 2019; Hickman et al., 2021; Léger-Goodes et al., 2022). Over half of child and adolescent psychiatrists in England report seeing CYP distressed about climate change and the environment (Royal College of Psychiatrists, 2020) and mental health professionals predict that they will increasingly see cases of mental health challenges caused or exacerbated by the climate crisis (Croasdale et al. 2023). In England, teachers report rising rates of both climate awareness and climate anxiety among their students, and students report climate change as one of the top three topics they worry about (Pearson, 2023). Strong psychological and emotional responses to awareness of the climate crisis and inaction of leaders are often rational, healthy, and adaptive, and should not be pathologized (Verplanken and Roy, 2013; Bhullar et al., 2022). However, without appropriate support, these responses can be ongoing stressors and negatively impact CYP’s mental health and wellbeing, affecting sleep and daily functioning, for example Hickman et al. (2021) and Ogunbode et al. (2021). CYP mental health is put even further at risk by feelings of betrayal from the adults in their lives and those in power (Hickman et al., 2021), by lack of opportunity to participate in decision making and for their voices to be heard, while also feeling the weight of responsibility for dealing with the climate crisis (Conner et al., 2023). Climate change is also a risk multiplier for mental health and compounds existing vulnerabilities, widening inequalities CYP already face (Lawrance et al., 2022). People with pre-existing mental health challenges, or those living in low income areas, for example, are at greater risk of harmful mental health impacts of climate change (Lawrance et al., 2022). Climate-related stress in early development may have damaging consequences such as increased risk of developing depression, anxiety, substance use disorders, and psychopathies later in life (Sheth et al., 2017; Wu et al., 2020). Direct experiences of climate impacts are linked to worsened symptoms and new cases of mental health challenges, and CYP can be highly vulnerable to these impacts. For example, experiences of flooding have been linked with post-traumatic stress disorder and behavioral problems in children (Stanke et al., 2012; Mort et al., 2016). These impacts will be felt more and more; children born today will face up to seven times as many extreme weather events as their grandparents (Thiery et al., 2021).

Growing emphasis on CCE in schools offers an opportunity for the mental health community to come alongside these efforts. Safeguarding mental health in the context of experiencing, having awareness of, and witnessing the impacts of the climate crisis is critical, particularly given that learning about climate change may exacerbate distress for CYP (Godsmark, 2020; British Science Association, 2023; Ojala, 2023). Government initiatives in England such as training senior mental health leads and expanding access to early mental health support aim to support schools to promote good mental health and wellbeing (HM Government, 2021). If addressing the harmful consequences that CCE (and climate change more widely) can have on CYP’s mental health is missed from these approaches, it puts at risk the success of initiatives to ensure schools are mentally healthy.

Integrating CCE with discussions about difficult emotions, mental health, psychological resilience, and healthy strategies for coping and acting also presents an opportunity to strengthen conversations around mental health in schools more broadly. CYP are a vital age group to target mental health support, with 75% of mental health challenges starting before age 18 (Kessler et al., 2005; HM Government, 2013). This age group is also experiencing rising mental health needs; in the United Kingdom rates of having a probable mental disorder rose from 1 in 9 children aged 7–16 in 2017 to 1 in 6 in 2020 (NHS Digital, 2023). Among those aged 17–19, rates rose from 1 in 10 in 2017 to 1 in 4 in 2022 (NHS Digital, 2023). CYP face multiple burdens on their mental health from compounding risks, including financial insecurity, lasting consequences of the COVID-19 pandemic (NHS Digital, 2023; Pearson, 2023), and climate change. In a 2023 survey, 61% of teachers in England reported seeing a rise in student anxiety around the issue of mental health and wellbeing over the past year (Pearson, 2023). Discussing difficult feelings in the context of the climate crisis might be easier, and come with less stigma, than talking about mental health more generally. For example, in England, a pilot study of a schools-based, co-created workshop to explore climate emotions enabled young people to make more sense of their thoughts and feelings about climate change. Discussions in the workshop also covered wider ground than just climate change and reflected the interconnected nature with other issues (Marks et al., 2023). This kind of tool could be a way into wider discussions on the many other pressures CYP face on their mental health.

## 3 How can mental health help achieve successful climate change education?

Successful CCE must appropriately inspire and equip young people to engage in a sustained way with the transformations of society toward a safer climate future [e.g., changing approaches to managing land and agriculture, building more cohesive communities (Shukla et al., 2022), and phasing out fossil fuels]. In that future, every career needs to be a “climate career,” as every aspect of our society will be affected by a changing climate, and will need to be part of the transformation to a greener world. Without equipping CYP with the right support and skills to engage with these very difficult topics and sustain action, the success of this mission is at risk, as is CYP’s mental health. Many young people who engage in climate activism report that a lack of support leads to burnout, and may be forced to step away from taking action on climate because of the toll on their mental health (Godden et al., 2021; Diffey et al., 2022; Conner et al., 2023). People working and engaging in climate spaces can be vulnerable to burnout, vicarious trauma and ultimately leaving these professions (Pihkala, 2020b). CCE must incorporate understanding of how CYP’s mental health and wellbeing can be affected by holding awareness of and learning about climate change, and what factors make engaging in climate action protective for mental health and wellbeing, versus which risk exacerbating harm. Not doing so risks causing further harm to an age group already vulnerable to experiencing the onset of poor mental health (Kessler et al., 2005; HM Government, 2013).

Taking climate action in sustainable ways requires appropriate support and skills in building resilience to do what can be psychologically challenging work. CCE also needs to incorporate ways to build this resilience, such as developing the skills to understand, reflect on and process their emotional responses, imagine the future they want to create, and build belief in their agency (Ojala, 2023). The skills needed to sustainably work in climate careers and engage in climate action (Cantell et al., 2019) also closely align with the skills needed to promote good mental health more generally—such as sitting with uncertainty, uncomfortable emotions, listening, communication, collaboration, self-care, and problem-solving.

Building these skills is not only needed, but wanted. Students in the United Kingdom report seeing school as a critical pathway to trusted information about climate change and climate action, with over 40% wanting to get more involved in climate action in schools (InterClimate Network, 2023). Young people are calling for solutions-oriented teaching that equips them with knowledge and skills to tackle the climate crisis and take control of their futures (British Science Association, 2023). By incorporating skill building for psychological resilience with climate education and student climate action, CYP can develop sustained capacity to live in a climate changed world and work toward a safer climate future, while promoting their ability to manage their own mental health.

## 4 Systemic barriers holding back integrating mental health into climate change education

Climate change education offers the opportunity to prepare CYP with the knowledge, tools, and skills for the future in ways that align inspiring climate action and safeguard mental health. Currently, systemic barriers challenge this opportunity. Teachers report that integrating more mentions of climate change into the national curriculum is what would most help CCE (Greer et al., 2023). Despite this, they report feeling unequipped to teach about climate change; 70% of United Kingdom surveyed educators say they have not received adequate training to educate students on climate change, its implications nor its solutions (Teach The Future, 2021). The impacts of climate change and benefits of climate action affect every part of societies, but just 17% of teachers say climate change is mentioned in core subjects outside of science and geography (Teach The Future, 2021). This lack of integrated approaches to CCE is compounded by lack of time and resources to develop ideas and approaches that can be delivered within an established and constrained curriculum, or CCE not being seen as a priority to school leadership teams (Pearson, 2023). Teachers also report that students becoming anxious about climate change can itself be a barrier to providing CCE, as can educators’ own feelings of uncertainty and overwhelm about how to deliver this education (Pearson, 2023).

Without appropriate pedagogical approaches to climate content, teachers lack confidence, awareness, knowledge, and support on how to hold the tension of protecting students against despair and worry while inspiring climate action (Education International and UNESCO, 2021). Teachers instead may have diverse and personal responses to students’ emotions about climate change, ranging from validating emotions, avoiding talking about emotion laden issues, or attempting to replace worry with hope (Ojala, 2023). Siloed approaches to strategies and funding for CCE and for mental health support is a barrier to implementing the support for educators that is needed. Educators themselves may also experience their own emotional responses to climate change and poor mental health that requires support.

## 5 Integrating mental health and climate change education in practice: learning from the whole school approach

### 5.1 What could integrated approaches look like?

Truly embedding and sustaining integration of mental health into CCE will require action from all parts of the education system: governments, educators, researchers, mental health experts, and parents and carers. This can learn from existing frameworks to achieve integrated, aligned, and sustained action in school settings, such a “whole school approach” (WSA). WSAs have been applied separately to both promoting good mental health and wellbeing, and for CCE. Mathie and Wals (2022) highlight that a WSA “seeks to re-orient [education] by anchoring it in different principles and values that contribute to education that is more responsive, relevant, responsible, and re-imaginative, in light of urgent global challenges.” The WSA to mental health and wellbeing encompasses commitment and action through the curriculum, leadership, early support for CYP, and relationships between students and staff (Anna Freud, 2023b). The WSA is endorsed by the Department for Education and National Institute for Health and Care Excellence in England (HM Government, 2021), and guidance and training is available to help schools implement these strategies (HM Government, 2021; Anna Freud, 2023a). While schools play a vital role in supporting young people’s mental health and wellbeing, teaching staff and school leaders are first and foremost educators and not clinically trained. Crucially, a WSA helps education staff to follow the appropriate steps to access wider support systems for CYP from mental health experts and services. Table 1 explores key features of the WSA and ways this could be adopted for integrating mental health into CCE.

**Table 1:** Eight principles of the whole school approach to promoting good mental health and wellbeing, and how these could be applied to practical integration of mental health into climate change education.

Principles of the whole school approach to mental health and wellbeing		A whole school approach in action for integrating mental health into climate change education
1 Leadership and management	Effective implementation of a WSA, and ensuring actions are integrated and sustained, requires a coordinated approach to supporting and championing the promotion of mental health and wellbeing.	Senior school leaders and governing bodies taking visible and tangible climate action, including putting in place sustainability leadership and climate action plans that involve and are well communicated to students and the whole school community. Seeing meaningful climate action being taken by leaders and societal actors can be protective against climate distress for CYP (Ojala, 2012; Diffey et al., 2022).
2 Curriculum teaching and learning	The development and promotion of resilience and support of social and emotional learning, through personal, social, health, and economic (PSHE) education and complemented through the wider curriculum.	Climate change integrated throughout the curriculum in ways that creatively inspire and equip CYP around climate change, create space for discussions around mental health, and help build psychological resilience. For example, this could explore how art, drama, music, creative writing, storytelling, and play can create ways for CYP to process their own emotional responses to climate change, and also creatively imagine the future they want to see and what role they want to play in building it.
3 Student voice	Involving students and providing opportunities to influence the decisions that impact them.	Opportunities for CYP to influence sustainability and climate action plans, such as establishing a student sustainability group which is consulted by senior leaders, and offering opportunities for students to take action on climate change within the school and wider community. CYP equipped with constructive narratives, coping strategies, and spaces where emotional responses to climate change can be heard, validated and where they are given the tools to reflect and process these responses and equip them for their futures.
4 Staff development	Promoting and supporting staff mental health and wellbeing is an important component of a WSA, enabling staff to support their students.	Staff trained and equipped to effectively deliver CCE and care for young people’s mental health and wellbeing, particularly where young people may be experiencing distress, and to refer and signpost CYP to mental health support. Crucially, staff must be in a position to listen to and validate the understandable emotions and distress young people are feeling about the facts of climate change and inaction of leaders. Providing support for staff’s own mental health and wellbeing, for example through supervision and reflection spaces, to understand their own emotional responses to climate change and witnessing the impacts of CCE on their students.
5 Identifying and monitoring impact	This allows education settings to understand the impact of the support put in place, and enables informed decision-making to respond to students’ mental health and wellbeing needs.	Adopting an evaluative approach to mental health and wellbeing, which encompasses climate change and associated school action and education to demonstrate the impact of integrated climate change and mental health education.
6 Parents and carers	Supporting and engaging parents and carers, trusted adults in CYP’s lives, to play a key role in supporting CYP mental health and wellbeing.	Schools working with parents and carers to provide resources and support, such as having conversations about climate change and supporting their families to take climate action.
7 Targeted support and appropriate referral	Supports early and effective intervention in ensuring poor mental health does not become entrenched.	Wider support system for CYP mental health (school nurses, counselors, and Mental Health Support Teams) equipped to recognize and respond to mental health and wellbeing needs arising from learning about climate change and experiencing climate impacts.
8 Ethos and environment	Includes the physical, social, and emotional environment where the school community spend the majority of their time and ensuring it promotes respect and values diversity.	CYP provided with the space and opportunity to reflect on their own responses to climate change, and understanding how they may regulate their emotions in response and be supported in actions they may wish to take. For example, spending time in, observing, and caring for nature within school grounds can be an important starting point for supporting both mental health and care for the environment.

The concept of a WSA to CCE and climate action in schools has also already been called for and implemented in approaches around the world (Gibb, 2016; European Commission, 2022; Mathie and Wals, 2022). While these approaches do not (to our knowledge) explicitly link with promoting good mental health, either in the context of climate change or more widely, some do encompass elements that align with this (e.g., building climate agency and community connection). New Zealand’s Education for Sustainability curriculum, for example, employs a WSA to sustainability and encompasses principles of systems thinking and the connected nature of the wellbeing of the planet and our own wellbeing (New Zealand Government Ministry of Education, 2023). Learnings could be drawn from across the ways a WSA has been implemented either to support mental health or CCE, to inform development of integrated approaches to both.

### 5.2 What is needed to achieve integrated approaches?

Working toward integrated approaches that promote and protect mental health and wellbeing and simultaneously maximize success of CCE requires building our understanding of what is needed, what works, and how to address systemic barriers. Key priorities for research, action, investment, and commitment are:

- *Building knowledge through research*. While the evidence base on how CYP’s mental health and wellbeing is affected by climate change is rapidly growing, key gaps in our knowledge remain on how these impacts are experienced (e.g., among younger age groups), and what support works, why and for who.
- *Co-design with CYP, teachers, school leadership teams, and parents and carers.* Exploring barriers and opportunities for implementing integrated CCE and mental health approaches and understanding what kinds of support can truly respond to the needs of CYP, teachers and school leaders can inform the development of evidence-based interventions that schools and policymakers can invest in.
- *Connecting, learning from, and evaluating existing tools*. Educators should be supported with guidance, frameworks, professional development, and case studies, with support from mental health and climate pedagogy experts. This should also encompass strategies and guidance for staff to support their own wellbeing and that of their peers. Work is already being done around the world to provide tools, resources and training to teachers and school leadership teams about CCE and mental health (Hoath and Dave, 2022; Climate Mental Health Network, 2023; Earthwatch Education, 2023; Force of Nature, 2023; Keep Northern Ireland Beautiful, 2023; Natural History Museum, 2023; Oxfam GB, 2023; ThoughtBox Education, 2023). Existing tools and resources need to be connected to each other to learn from, standardize approaches, and inform best practice.
- *Addressing siloes in investment around CCE and mental health support in schools*. Ultimately, coordinated government investment is critical to support an effective approach and cost efficiency. There is great work already happening in the realms of mental health and wellbeing in schools and CCE, however they are being treated entirely individually, whereas quick wins could be achieved by embedding integrated approaches into these existing initiatives. For example, linking CCE and the risk of distress and anxiety through to the senior mental health leads in England and mental health support teams will enable schools to signpost and refer appropriately.

## 6 Discussion

Children and young people (CYP) face a present and future that is uncertain and frightening, and while decision makers delay sufficient climate action, the mental health and wellbeing of CYP is at risk. Young people see school as a critical pathway to trusted information about climate change and equipping them to take climate action (InterClimate Network, 2023). Currently, however, systemic barriers in education risk turning young people away from climate action and harming their mental health and wellbeing in the context of learning about climate change. By bringing mental health and wellbeing into CCE, we can better achieve the important outcomes of preparing children and young people to live in an uncertain future and take sustainable climate action. We can also foster wider conversations, awareness and support for mental health. Existing methods to achieve aligned and sustained action in schools, such as a whole-school approach, could provide a framework that could help achieve such integration. To inform and enact integrated approaches, research, co-design, investment, and commitment is needed from across the education, mental health and climate change communities, accompanied by coordinated government support. As awareness of climate change and associated distress increases, poor mental health among CYP rises, and attention on CCE grows, this is a critical moment for aligned action. CYP must be, and want to be, supported to foster good mental health and develop the skills and coping strategies to not only live with the uncertainty that our present and future world holds, but also take control of their futures.

## Data availability statement

The original contributions presented in the study are included in the article/supplementary material, further inquiries can be directed to the corresponding author.

## Author contributions

JNLV: Conceptualization, Writing – original draft, Writing – review & editing. AC: Writing – review & editing, Writing - original draft. LS: Writing – review & editing. HD: Writing – review & editing. LH: Writing – review & editing. EL: Conceptualization, Writing – review & editing.
